# LSTM-Based Absolute Position Estimation of a 2-DOF Planar Delta Robot Using Time-Series Data

**DOI:** 10.3390/s26020470

**Published:** 2026-01-10

**Authors:** Seunghwan Baek

**Affiliations:** Department of Electrical and Electronic Engineering, Youngsan University, Yangsan 50510, Republic of Korea; shbaek0@gmail.com

**Keywords:** LSTM, robot dynamics, robot manipulability, 2-DOF delta robot, time-series data

## Abstract

Accurately estimating the absolute position of robots under external loads is challenging due to nonlinear dynamics, posture-dependent manipulability, and structural sensitivities. This study investigates a data-driven approach for absolute position prediction of a 2-DOF planar delta robot by learning time-series force signals generated during manipulability-driven free motion. Constant torques of opposite directions were applied to the robot without any position or trajectory control, allowing the mechanism to move naturally according to its configuration-dependent manipulability. Reaction forces measured at the end-effector and relative encoder variations were collected across a grid of workspace locations and used to construct a 12-channel time-series input. A hybrid deep learning architecture combining 1D convolutional layers and a bidirectional LSTM network was trained to regress the robot’s absolute X–Y position. Experimental results demonstrate that the predicted trajectories closely match the measured paths in the workspace, yielding overall RMSE values of 3.81 mm(X) and 2.94 mm(Y). Statistical evaluation using RMSE shows that approximately 83.73% of all test sequences achieve an error below 5 mm. The findings confirm that LSTM models can effectively learn posture-dependent dynamic behavior and force-manipulability relationships.

## 1. Introduction

In robot control, sensor signals, joint positions, and force/torque information are all time-varying data, and the current state is strongly dependent on past movements. Accurately understanding a robot’s actual motion and predicting its future behavior based on such time series is essential in various applications, including high-precision tasks, safe human–robot interaction, rehabilitation, and medical robotics. However, traditional statistical models or static regression approaches are limited in capturing temporal dependencies, nonlinear patterns, and the diverse uncertainties that arise in real robot environments.

In recent years, deep learning-based models have been widely adopted in time series forecasting to address complex dynamic patterns. Kim et al. [1] reported that architectures such as LSTM, TCN, and Transformers have become increasingly sophisticated for time series analysis, emphasizing the importance of modeling long-term dependencies. Li and Law [2] pointed out that the linearity and stationarity assumptions of ARIMA-type models are inadequate for complex nonlinear time series, while deep learning effectively overcomes these limitations. Similarly, Kong et al. [3] highlighted automatic feature extraction and nonlinear learning as key strengths of deep learning-based approaches.

Among these methods, Long Short-Term Memory (LSTM) networks are particularly effective for real-world time series data that are nonlinear and noisy, owing to their gate-based structure that enables simultaneous learning of short- and long-term temporal information. Lindemann et al. [4] analytically demonstrated that LSTM outperforms conventional RNNs in temporal stability and memory retention, while Mahmoudi [5] showed that LSTM can sensitively capture patterns even in chaotic dynamic systems, making it well-suited for robot systems with strong dynamic characteristics.

In the robotics domain, the need for data-driven approaches is particularly high. Recent studies have demonstrated that intelligent and adaptive control strategies, such as fuzzy sliding mode control, can effectively handle nonlinearities and uncertainties in robotic manipulators [6]. Despite these advances, actual robot motions involve various uncertainties, including joint clearance, friction, compliance, and sensor noise, making it difficult for purely physics-based inverse dynamics models to accurately reflect real behavior. As a result, recent robot control research has increasingly explored hybrid approaches combining model-based and data-driven methods, or purely data-driven models for motion prediction and compensation. Neural-network-based methods have been applied to robot calibration and pose estimation to compensate for geometric and non-geometric errors that are difficult to model analytically [7,8,9]. Vagale et al. [10] experimentally demonstrated that LSTM achieves high performance in short-term sensor prediction for mobile robots, even in the presence of disturbances and measurement noise.

These deep learning techniques, including LSTM and BiLSTM architectures, have been successfully employed to capture nonlinear temporal relationships in robot dynamics, energy consumption, and rehabilitation-related motion patterns [11,12,13,14]. Liu et al. [15] developed an LSTM-based inverse dynamics model for a 7-DOF hydraulic manipulator and showed that accurate torque prediction is possible even when using only joint position inputs, indicating that LSTM can effectively capture nonlinear temporal patterns in robot motion. Rueckert et al. [16] further demonstrated that LSTM can efficiently process large-scale time series data and learn complex inverse dynamics more effectively than Gaussian Processes or LWPR. Reuss et al. [17] proposed a hybrid inverse dynamics framework combining analytical models with LSTM-based residual learning, showing improved compensation for friction and unmodeled effects. In addition, Zhang et al. [18] demonstrated real-time prediction of external torques in a 6-DOF robot for collision detection, while Huang et al. [19] showed that advanced LSTM variants with spatiotemporal attention can further enhance inverse dynamics prediction accuracy.

Beyond robotics, hybrid deep learning models combining LSTM with advanced sequence-learning frameworks have shown strong robustness in complex and noisy time-series forecasting tasks [20], while alternative data-driven methods such as dynamic mode decomposition have been proposed for near-periodic systems [21].

Although absolute position estimation in robotic systems is typically achieved through forward kinematics using absolute encoders and known kinematic parameters, there exist practical scenarios in which such information becomes unreliable or unavailable. These include temporary loss or failure of absolute encoders, unknown or time-varying kinematic parameters due to mechanical wear or structural compliance, and rehabilitation or human–robot interaction systems where external forces and load conditions significantly affect the robot’s behavior. In such cases, force sensor signals and relative motion information provide complementary cues that implicitly reflect posture-dependent dynamics and interaction-induced responses, which are not directly observable through kinematics alone.

In this context, the objective of this study is not to replace conventional forward kinematics when accurate absolute encoder information is available, but rather to investigate whether absolute position information can be inferred from force-based time-series data and relative encoder signals during torque-driven free motion. By leveraging manipulability-driven motion patterns and the dynamic signatures embedded in reaction-force signals, the proposed approach aims to estimate the robot’s absolute position under conditions where classical kinematic reconstruction is insufficient or degraded.

Based on the above practical considerations, the approach of this study has clear validity for several reasons. First, most prior LSTM-based robot prediction studies have focused on torque estimation or model-based inverse dynamics compensation, whereas few studies have directly measured and predicted the real-time force responses generated when a robot is subjected to external loads. Second, by attaching a force sensor to the end-effector and applying different weights, nonlinear structural deformation, load-dependent responses, and dynamic uncertainties within the robot mechanism are naturally reflected in the data. The characteristics that cannot be captured using theoretical torque calculations or idealized simulation data. Third, learning these real force-based time series using LSTM offers practical advantages by complementing inherent nonlinearities, external force responses, and structural sensitivities that cannot be fully described by physical models. In this study, force sensor data were collected from a 2-DOF planar delta robot under various load conditions, preprocessed and normalized, and used to train an LSTM model that predicts future force patterns and robot behavior. Thus, this work goes beyond model-based approaches or simulation-level validation and represents one of the first attempts to verify LSTM predictive performance using actual force-time sequences measured in a robot with externally applied loads. The results provide meaningful evidence supporting the use of such models for robot position correction, state estimation, and dynamic behavior stabilization in practical robotic applications.

## 2. Configuration of the Robot System

**Figure 1:**
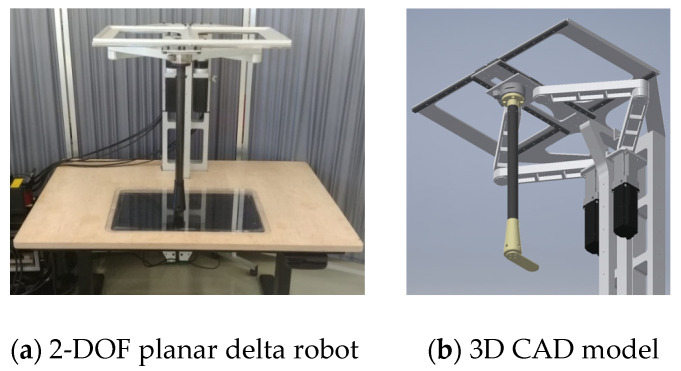
2-Axis delta(parallel) robot for upper-limb rehabilitation.

The robot used in this study is a 2-DOF planar delta(parallel) robot developed for upper-limb rehabilitation, designed to provide precise planar movements required for repetitive rehabilitation exercises. The overall design philosophy, development process, safety architecture, and detailed mechanical configuration of the robot have been comprehensively described in the authors’ previous work, which is currently under review. In this paper, only the elements directly relevant to the LSTM-based behavior prediction are summarized. The overall structure of the robot is presented in Figure 1.

### 2.1. Robot Kinematics

The robot is a planar parallel mechanism composed of two rotational actuators (*θ*_1_ and *θ*_4_) positioned on the left and right sides, along with the corresponding upper and lower links (*L*_1_, *L*_2_/*L*_3_, *L*_4_) connected to them. The lengths of the links are fixed, and the position of the end-effector (*x*, *y*) is determined by the geometric constraints formed by the two links, which can be expressed as the intersection of two circles.

**Figure 2:**
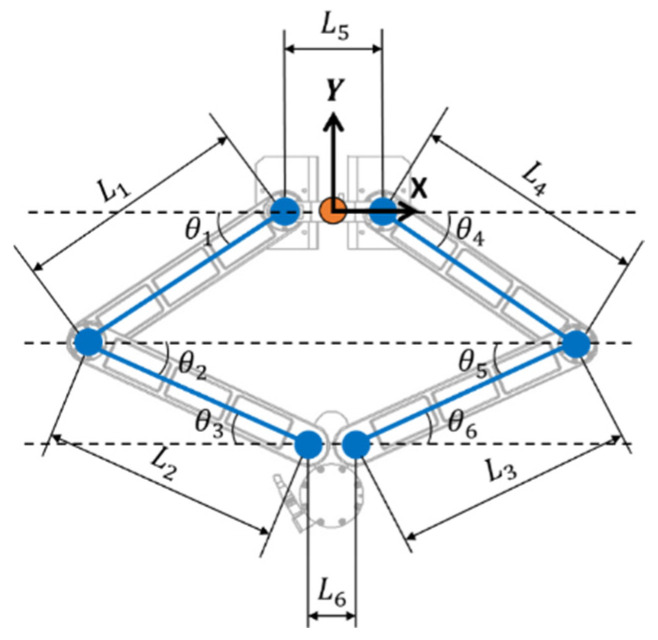
Kinematic structure of the 2-Axis delta(parallel) robot.

The kinematic structure of the 2-axis delta robot is depicted in Figure 2, where *θ*_1_ and *θ*_4_ are the joint angles driven by the motors. The remaining joint angles *θ*_2_, *θ*_3_, *θ*_5_, *θ*_6_ are determined by the closed-loop mechanism formed by *θ*_1_ and *θ*_4_. The distances between the joints, denoted as *L*_1_ to *L*_4_, define the kinematic relationships. *L*_1_ to *L*_4_ represent the distances between the rotational centers of each joint in the robot, and they also correspond to the lengths of each arm. *L*_5_ denotes the distance between the driving motors, while *L*_6_ indicates the distance between the joints of the end-effector.

The mathematical model for robot kinematics can be defined as follows.

(1) $$-L_{1}cos\theta_{1}+L_{2}cos\theta_{2}+L_{6}+L_{3}cos\theta_{5}-L_{4}cos\theta_{4}-L_{5}=0$$

(2) $$-L_{1}sin\theta_{1}-L_{2}sin\theta_{2}+L_{3}sin\theta_{5}+L_{4}sin\theta_{4}=0$$

(3) $$P_{x}=-\frac{1}{2}L_{5}-L_{1}cos\theta_{1}+L_{2}cos\theta_{2}+\frac{1}{2}L_{6}$$

(4) $$P_{y}=-L_{1}sin\theta_{1}-L_{2}sin\theta_{2}$$

(5) $$P_{x}=\frac{1}{2}L_{5}+L_{4}cos\theta_{4}-L_{3}cos\theta_{5}-\frac{1}{2}L_{6}$$

(6) $$P_{y}=-L_{4}sin\theta_{4}-L_{3}sin\theta_{5}$$

By Equations (3)–(6), we can obtain equations:

(7) $$A_{1}+B_{1}cos\theta_{1}-C_{1}sin\theta_{1}+D_{1}=0$$

(8) $$A_{2}+B_{2}cos\theta_{4}-C_{2}sin\theta_{4}+D_{2}=0$$

Here,

(9) $$A_{1}=P_{x}^{2}+P_{y}^{2}+L_{1}^{2}-L_{2}^{2}+0.25L_{5}^{2}+0.25L_{6}^{2}\;$$

(10) $$B_{1}=2P_{x}L_{1}+L_{1}L_{5}-L_{1}L_{6}\;$$

(11) $$C_{1}=2P_{y}L_{1}\;$$

(12) $$D_{1}=P_{x}L_{5}+P_{x}L_{6}-0.5L_{5}L_{6}\;$$

(13) $$A_{2}=P_{x}^{2}+P_{y}^{2}+L_{4}^{2}-L_{3}^{2}+0.25L_{5}^{2}+0.25L_{6}^{2}$$

(14) $$B_{2}=-2P_{x}L_{1}+L_{4}L_{5}-L_{4}L_{6}\;$$

(15) $$C_{2}=2P_{y}L_{4}$$

(16) $$D_{2}=-P_{x}L_{5}+P_{x}L_{6}-0.5L_{5}L_{6}$$

After employing trigonometric identities, the equations are rearranged for *θ*_1_ and *θ*_4_.

(17) $$\theta_{1}=2{tan}^{-1}\left(\frac{-B_{1}\pm \sqrt{B_{1}^{2}-(A_{1}-C_{1}+D_{1})(A_{1}+C_{1}+D_{1})}}{\left(A_{1}-C_{1}+D_{1}\right)}\right)$$

(18) $$\theta_{4}=2{tan}^{-1}\left(\frac{-B_{2}\pm \sqrt{B_{2}^{2}-(A_{2}-C_{2}+D_{2})(A_{2}+C_{2}+D_{2})}}{\left(A_{2}-C_{2}+D_{2}\right)}\right)$$

Here, x and y denote the coordinates of the end-effector in the X–Y plane. Because of the closed-loop configuration of the parallel robot, the kinematic relationships among the joints provide consistent solutions regardless of which of the two derived equations is applied. The actuated joints controlling the robot are *θ*_1_ and *θ*_4_; hence, the inverse kinematics are defined through equations that map these joint angles to the end-effector position.

### 2.2. Drive System

The actuation system consists of two 400 W AC servo motors paired with precision gear reducers with a ratio of 10:1, providing sufficient force and accuracy for the repetitive motions required in rehabilitation tasks. Each motor is equipped with a high-resolution absolute encoder, enabling accurate computation of the end-effector position and serving as a reference for kinematics-based position tracking. In this study, the position and state data obtained from these actuation and sensing components were used as reference signals for training and evaluating the LSTM-based prediction model.

### 2.3. Configuration for End-Effector and Force Sensor

The robot incorporates a force sensor as an essential component for upper-limb rehabilitation, enabling quantitative measurement of both active and passive patient movements. This sensor was not added specifically for the purposes of this study; rather, it has been an integral part of the system since the initial design stage. A two-axis force sensor mounted on the end-effector measures reaction forces in the *Fx* and *Fy* directions, allowing real-time monitoring of user resistance, muscle tone variations, and external force interactions.

**Figure 3:**
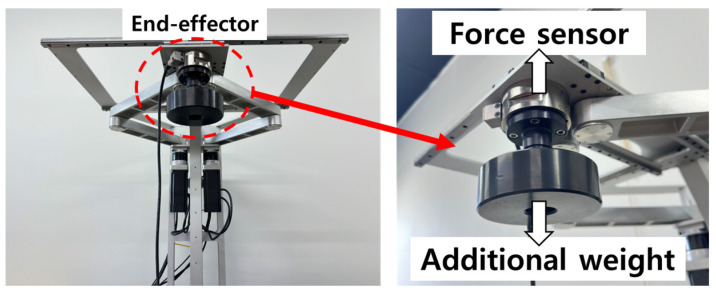
Detail view of the robot end-effector.

In this study, this originally embedded force measurement device was utilized to extract time-series data representing dynamic variations in the parallel mechanism and corresponding reaction force patterns under different loading conditions with additional weights attached to the end-effector. The nonlinear and structure-dependent characteristics observed through this process constitute the essential dynamic patterns that the LSTM model aims to learn. The configuration of the end-effector and the integrated force sensor is illustrated in Figure 3.

### 2.4. Robot Manipulability

The 2-DOF planar delta robot used in this study generates characteristic motion patterns even without explicit position control commands. When arbitrarily chosen constant motor torque is applied, the resulting movement naturally follows the manipulability characteristics determined by the robot’s kinematic structure. This behavior emerges from the Jacobian configuration, link arrangement, and geometric constraints inherent to the parallel mechanism. Consequently, even without specifying a target position, the robot moves in the direction that is most favored and easiest to achieve according to the manipulability distribution associated with its current posture.

In particular, the manipulability ‘*w*’ defined by Yoshikawa [22] is based on the singular values of the Jacobian ‘*J*’, and a larger value indicates that the end-effector can move more easily in a specific direction. While large singular values indicate directions in which the end-effector can easily generate velocity, they simultaneously correspond to directions where the robot transmits external forces less effectively. Conversely, smaller singular values represent directions in which external forces are transmitted more efficiently to the joints. Thus, the principal axes of the force manipulability ellipsoid characterize the directions of strong or weak force transmission within the mechanism.

According to the study by Wu et al. [23], a 2-DOF planar delta robot exhibits the most uniform manipulability and dynamic manipulability near the center of its workspace, while nonlinear characteristics increase toward the boundaries due to the geometric configuration of the links. These structural characteristics also apply to the robot used in this study, resulting in distinct reaction-force patterns and dynamic responses at different positions, even under the same torque input.

To mathematically characterize the manipulability properties described above, the kinematic relationships of the robot must be explicitly formulated. Based on the kinematic structure illustrated in Figure 2, the Jacobian matrices of each arm and their combined task-space representation for the planar delta robot used in this study are derived as follows.

Each arm of the robot is a two-link planar manipulator. Accordingly, the Jacobian matrix *J_R_*(*θ*_1_, *θ*_2_) describing the mapping between the joint angles (*θ*_1_, *θ*_2_) of the right arm and the end-effector position (*x*, *y*) can be written as:

(19) $$J_{R}(\theta_{1},\theta_{2})=\left(\begin{array}{cc} -L_{1}sin\theta_{1}-L_{2}sin(\theta_{1}+\theta_{2}) & -L_{2}sin(\theta_{1}+\theta_{2})\\ L_{1}cos\theta_{1}+L_{2}cos(\theta_{1}+\theta_{2}) & L_{2}cos(\theta_{1}+\theta_{2}) \end{array}\right)$$

The Jacobian of the left arm has the same structure and is given by:

(20) $$J_{L}(\theta_{4},\theta_{5})=\left(\begin{array}{cc} -L_{4}sin\theta_{4}-L_{3}sin(\theta_{4}+\theta_{5}) & -\;L_{3}sin(\theta_{4}+\theta_{5})\\ L_{4}cos\theta_{4}+L_{3}cos(\theta_{4}+\theta_{5}) & L_{3}cos(\theta_{4}+\theta_{5}) \end{array}\right)$$

Because the two arms simultaneously constrain the end-effector in a parallel configuration, the overall task-space Jacobian of the robot is approximated in this study by averaging the two individual Jacobians:

(21) $$J(q)=\frac{1}{2}\left(J_{R}\left(\theta_{1},\theta_{2}\right)+J_{L}(\theta_{4},\theta_{5})\right)$$

where $J(q)\in R^{2\times 2}$ satisfies the relationship between the joint velocity vector $\dot{q}$ and the end-effector velocity $\dot{x}$:

(22) $$\dot{x}=J(q)\dot{q}$$

The manipulability ellipsoid is computed by performing eigenvalue decomposition on $JJ^{⊤}$, where the resulting eigenvalues and eigenvectors determine the ellipsoid’s principal axes and orientation.

(23) $$JJ^{T}=J(q){J(q)}^{T}$$

The Jacobian formulation adopted here is a simplified approximation used to illustrate posture-dependent manipulability trends under free-motion conditions. Since the proposed framework relies on measured force responses rather than exact analytical Jacobians, this approximation is adequate for the present study.

**Figure 4:**
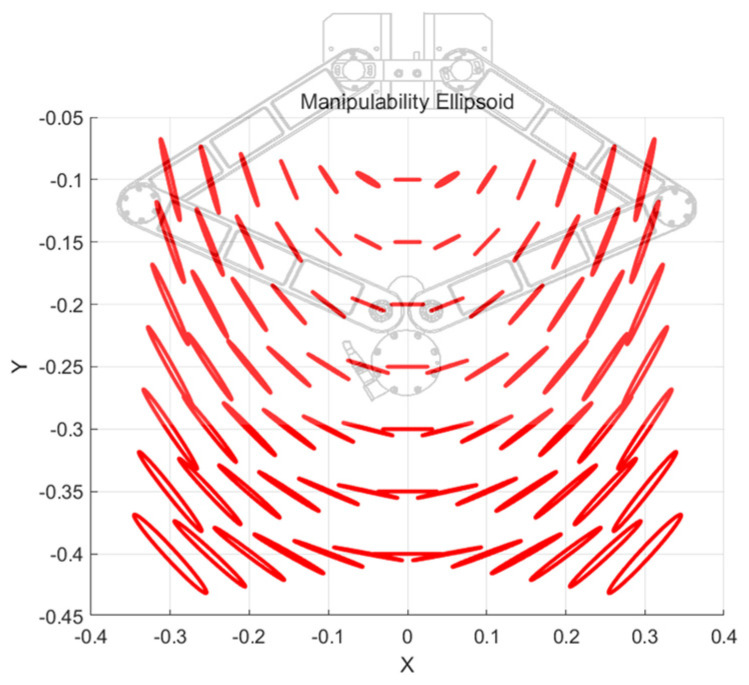
Manipulability in the range of motion.

Figure 4 illustrates the distribution of force manipulability across the robot’s reachable workspace. The ellipsoids were computed over the feasible region, spanning approximately from −0.30 to 0.30 m in the X direction and from −0.40 to −0.10 m in the Y direction. Within this area, each ellipsoid represents how effectively an external force applied at the end-effector is transmitted to the joints at that specific posture.

In the central portion of the workspace the ellipsoids appear relatively balanced and moderately sized, indicating that external forces are transmitted more uniformly and with higher sensitivity. Toward the outer boundaries, however, the ellipsoids become elongated and skewed, revealing directions in which force transmission becomes significantly less effective due to geometric constraints. These posture-dependent differences highlight the inherent dynamic characteristics of the planar parallel mechanism.

In this study, such manipulability-based and position-dependent dynamic characteristics were utilized to collect time-series data from the force sensor. Even with a constant torque input, the robot naturally generates free motion determined by its manipulability properties, and the resulting reaction forces measured at the end-effector reflect structural nonlinearities, inertial variations, and differences in dynamic manipulability. This manipulability-driven motion enabled the collection of reaction-force data under various loading conditions, which in turn provided a dataset allowing the LSTM model to learn both the observable force responses and the inherent dynamic characteristics of the robot.

## 3. LSTM Network Architecture and Learning Framework

### 3.1. Overview of the LSTM Network

The Long Short-Term Memory (LSTM) network is a type of recurrent neural network (RNN) designed to effectively learn both short- and long-term dependencies in time-series data. An LSTM cell consists of an input gate, a forget gate, and an output gate, which collectively regulate the flow of information. Through this gated architecture, the network retains important temporal features while naturally discarding irrelevant or redundant information.

In this study, the LSTM model was adopted to predict the force time-series signals (*Fx* and *Fy*) measured at the robot’s end-effector. These signals inherently contain characteristics of the robot’s internal dynamics, variations due to external loading, and posture-dependent force manipulability.

### 3.2. LSTM-Based Learning Model Architecture

The LSTM-based learning model was designed to predict the absolute position of the robot by receiving force-sensor signals measured at the end-effector as inputs. To accomplish this, the experimental data were first segmented according to repeated motion cycles, and each segment was interpolated onto a common time axis to construct fixed-length time-series sequences. Based on the measured force signals (*Fx*, *Fy*), additional auxiliary features such as moving averages, cumulative sums, and first-order differences were generated, and the relative changes in the encoder readings as well as cumulative encoder displacements were included, resulting in a total of 12 input channels. The outputs were defined as the robot’s absolute position (*X*, *Y*) at each time step, and both inputs and outputs were normalized using the mean and standard deviation computed from the training dataset.

The LSTM model architecture begins with a sequence input layer, followed by a feature-extraction module composed of one-dimensional convolutional layers, batch-normalization layers, and ReLU activation functions. A bidirectional LSTM layer is then employed to capture temporal dependencies and nonlinear patterns within the data. Finally, a fully connected layer generates the two-dimensional regression outputs corresponding to the predicted positions (*X*, *Y*).

**Figure 5:**
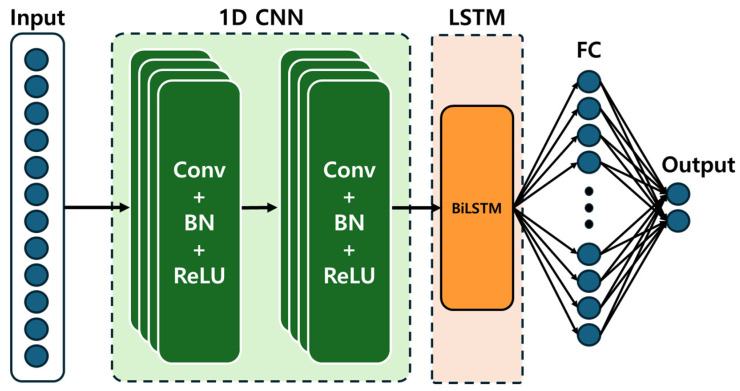
LSTM-Based Learning Model Architecture.

The network architecture shown in Figure 5 is organized as follows:

- Input layer
  Input dimension: 12 channels
- Two 1D convolutional layers
  Filter size: 5
  Number of filters: 36
  Padding: “same”
- Batch normalization and ReLU activation
- BiLSTM layer
  Number of hidden units: 64
  Output mode: sequence
- Fully connected layers (FC)
  Hidden layer → ReLU → Output layer (2-dimensional X, Y prediction)
- Regression layer

Model training was performed using the Adam optimizer, with an initial learning rate of $3\times 10^{-4}$, a mini-batch size of 6, and a sequence length of 256. A learning-rate decay schedule and gradient clipping were applied to ensure stable training performance.

- Training hyperparameters:
- Initial learning rate: $3\times 10^{-4}$
- Mini-batch size: 6
- Hidden units: 64
- Epochs: 100
- Gradient threshold: 1
- Shuffle: every-epoch

### 3.3. Definition of Input and Output Data

The input data consist of time-series measurements obtained from the force sensor mounted at the end-effector and joint encoders, forming a total of 12 channels. The primary force signals are the measured reaction forces *Fx* and *Fy*, and several auxiliary channels were derived to facilitate pattern learning in the LSTM network. Additionally, relative encoder values from each actuator were included as supplementary signals, providing information on joint motion even though they do not directly represent absolute position.

- Primary force signals (2 channels)
- $F_{x}(t)$: Reaction force along the *x*-axis
- $F_{y}(t)$: Reaction force along the *y*-axis
- Auxiliary feature channels (6 channels)
- MA($F_{x}$), MA($F_{y}$): Moving average (low-pass filtered) signals
- CUM($F_{x}$), CUM($F_{y}$): Cumulative sums (integration-like features)
- DIFF($F_{x}$), DIFF($F_{y}$): First-order differences (derivative-like features)
- Encoder-based auxiliary signals (4 channels)
- Enc1(t), Enc2(t): Relative joint encoder values
- Enc1_comsum_(t), Enc2_comsum_(t): Cumulative sums of incremental encoder changes

Although relative encoder signals are included as part of the input, they do not provide absolute position information and cannot be directly used to recover position through forward kinematics. Instead, they serve as auxiliary factors representing repeatable joint motion patterns during manipulability-driven free motion. The primary source of absolute position inference in the proposed framework arises from force-based dynamic responses, while the encoder signals provide complementary temporal context rather than explicit geometric information.

The outputs are the robot’s absolute positions X(t) and Y(t) at each time step. Both output channels are normalized and predicted through regression in the LSTM model. In this study, the proposed LSTM framework is trained to directly estimate the robot’s absolute positional trajectories from force-based time-series data.

## 4. Experimental Setup and Data Collection

The experiment designed to observe the dynamic characteristics of the robot began by positioning the robot at an arbitrary posture and applying constant torques of equal magnitude but opposite direction to the two drive motors for a fixed duration. Position control or trajectory tracking was not employed; instead, the experiment focused exclusively on observing the free motion generated by torque inputs within the parallel mechanism. Under these conditions, the 2-DOF planar delta robot naturally exhibits characteristic motion patterns determined by its manipulability.

Considering that manipulability imposes a configuration-dependent tendency in the robot’s motion, we assumed that identical torque inputs would produce similar motion ranges and relative encoder variations for each initial posture. For this reason, the relative encoder signals were included as auxiliary inputs to capture consistent dynamic behavior across repeated trials although not offering absolute position information.

To construct the training dataset, experiments were conducted by placing the robot at various initial positions. The training region spanned from 180 to −180 mm along the *X*-axis and from −380 to −240 mm along the *Y*-axis, with grid points spaced at 20 mm intervals. At each position, identical torque inputs were applied to the robot, and the resulting reaction forces from the free motion of the attached weight were recorded 50 times to capture subtle variations and environmental disturbances.

In contrast, the test dataset was collected from a position range independent of the training set. The test region covered 170 to −170 mm along the *X*-axis and −370 to −250 mm along the *Y*-axis, again with 20 mm spacing. Thus, although the test area is contained within the overall boundaries of the training region, it does not share any identical sampling points. This design allows for evaluating the LSTM model’s ability to generalize and predict robot behavior from initial positions not included in the training set.

## 5. Test Results and Discussions

**Figure 6:**
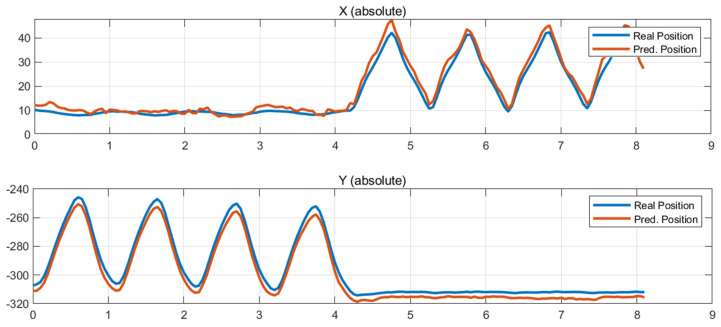
Test results at (10, −310).

**Figure 7:**
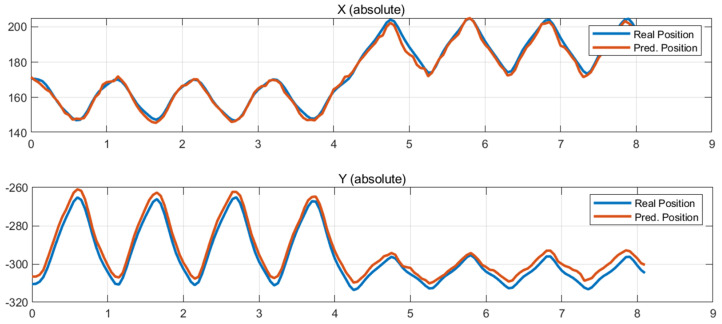
Test results at (170, −310).

**Figure 8:**
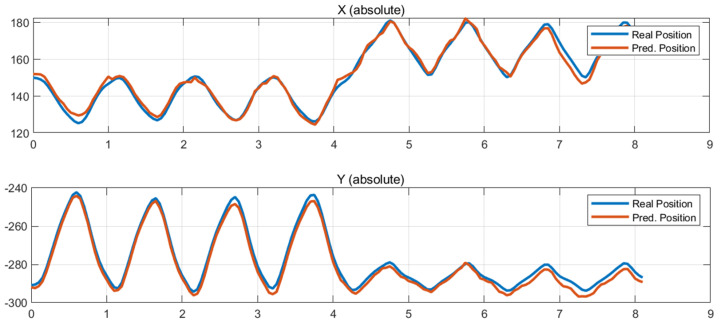
Test results at (−170, −310).

Figure 6, Figure 7 and Figure 8 present some comparisons between the predicted position trajectories by the LSTM model obtained from force-based input signals at some initial robot positions and the real measurements. The selected plots represent locations chosen to capture different levels of operational difficulty, including the central region of the workspace, the left and right outer boundaries.

The predicted trajectories closely match the measured trajectories, demonstrating that the LSTM model effectively learned the robot’s nonlinear dynamic characteristics, force manipulability, and load-dependent reaction-force patterns.

Figure 6 presents the prediction results for an initial posture located near the center of the workspace, such as the region around (X, Y) = (10 mm, −310 mm). Figure 7 and Figure 8 present the prediction results obtained at the right and left outer regions of the workspace, corresponding to the initial positions (X, Y) = (170 mm, −310 mm) and (−170 mm, −310 mm), respectively.

The measured and predicted position trajectories almost overlap, showing consistently small errors in both the X and Y directions throughout the entire time sequence. In the central region of the workspace, where the robot exhibits structurally stable dynamics, the model accurately captures the motion pattern and reproduces smooth trajectories. Even in the outer regions, where nonlinearity increases and dynamic manipulability decreases, the predicted trajectories follow the detailed variations in the measured data with sufficiently reliable performance. These results indicate that the LSTM model successfully is adapted to the position-dependent variations and structural sensitivities of the robot, thereby achieving robust generalization capability.

For all experimental locations, the prediction accuracy was quantified using the Root Mean Squared Error (RMSE) and is summarized statistically in Table 1. And also the ablation study of prediction accuracy is defined in Table 2.

The RMSE is defined as

(24) $$RMSE=\sqrt{\frac{1}{N}\sum_{i=1}^{N}(y_{i}-{\hat{y}}_{i}{)}^{2}}$$

Which measures the square root of the squared average difference between the actual values and the predicted values. The RMSE is one of the most widely used performance metrics for evaluating LSTM-based regression models.

In this study, the RMSE was computed for the X and Y axes of each test sequence, and the proportion of samples whose RMSE fell below threshold values ranging from 1 mm to 6 mm was statistically summarized. Among all test sequences, approximately 6.75% exhibited an RMSE below 1 mm, 28.97% below 2 mm, and 49.61% below 3 mm. When the threshold was extended to 4 mm and 5 mm, 71.43% and 83.73% of the samples, respectively, fell within these error bounds. Ultimately, about 90.08% of all test sequences showed an RMSE of 6 mm or less.

From an axis-wise perspective, the X-direction tended to exhibit slightly larger prediction errors, whereas the Y-direction demonstrated more stable performance, with a higher proportion of samples falling into the lower-RMSE ranges. The overall absolute RMSE was 3.81 mm for the X direction and 2.94 mm for the Y direction. In addition, an ablation study revealed that using only force sensor inputs resulted in errors RMSE of 5.59 mm in X and 5.30 mm in Y, the overall motion trends have followed the robot’s actual behavior across the workspace. Furthermore, incorporating encoder-based auxiliary inputs contributed to improved prediction accuracy, demonstrating that the combination of force signals and relative joint information enhances the model’s ability to estimate absolute position more precisely.

These results indicate that the proposed LSTM-based prediction model provides generally reliable performance for position estimation across the robot’s entire workspace. In particular, despite the workspace containing regions of near-uniform manipulability as well as boundary areas with strong kinematic nonlinearities, the model maintained stable prediction errors within only a few millimeters. However, applications requiring precision of sub-millimeter level in absolute position calibration may necessitate additional correction techniques or enhanced model architectures. Nevertheless, the experimental results confirm that the LSTM model is effective for force sensor–based motion prediction and absolute position estimation of the robot.

## 6. Conclusions

This study presented the LSTM-based framework for estimating the absolute position of a 2-DOF planar delta robot using force time-series data generated under manipulability-driven free motion. By applying constant torques without position control, the robot produced posture-dependent motion patterns governed by its manipulability, allowing the force sensor to capture nonlinear structural responses and load-induced dynamics. A 12-channel input which comprising force signals, auxiliary temporal features, and relative encoder variations was used to train a hybrid CNN–BiLSTM model. Experimental validation across both central and peripheral workspace regions demonstrated that the predicted trajectories closely followed the measured paths, despite increased nonlinearities near workspace boundaries. The RMSE analysis also showed that the model provides stable, few millimeter-level prediction accuracy, suggesting that it effectively learns the robot’s configuration-dependent dynamic behavior.

Future work will focus on improving prediction precision, particularly for applications requiring sub-millimeter accuracy. Additional studies will investigate how variations in external weights and applied torques influence force responses and prediction robustness. Finally, we aim to extend the proposed framework toward absolute-position recovery when the robot loses its reference state, enabling data-driven recalibration and drift compensation in long-term autonomous robotic systems.

Furthermore, from a data-driven modeling perspective, future studies will include systematic comparisons with standard baseline approaches such as RNNs and MLPs to more clearly quantify the strengths and limitations of the proposed force-based LSTM framework.

## Figures and Tables

**Table 1:** Summary of LSTM predictions.

RMSE threshold	Total Score (%)	X Score (%)	Y Score (%)
1 mm ≤ RMSE	6.75	0.00	13.49
2 mm ≤ RMSE	28.97	22.22	35.71
3 mm ≤ RMSE	49.61	43.65	55.50
4 mm ≤ RMSE	71.43	69.05	73.81
5 mm ≤ RMSE	83.73	77.78	89.68
6 mm ≤ RMSE	90.08	86.51	93.65

**Table 2:** Ablation study for LSTM (RMSE).

LSTM Input	X-RMSE (mm)	Y-RMSE (mm)
$2\;\mathrm{inputs}:\;F_{x}(t)$ $,\;F_{y}(t)$	5.59	5.30
$4\;\mathrm{inputs}:\;F_{x}(t)$ $,\;F_{y}(t)$ , Enc1(t) , Enc2(t)	4.28	3.08
12 inputs: $F_{x}(t)$, $F_{y}(t)$, MA($F_{x}$), MA($F_{y}$), CUM($F_{x}$), CUM($F_{y}$), DIFF($F_{x}$), DIFF($F_{y}$), Enc1(t), Enc2(t), Enc1_com_(t), Enc2_com_(t)	3.81	2.94

## Data Availability

The raw data supporting the conclusions of this article will be made available by the authors on request.
